# Naturally Acquired Rabies in White-Eared Opossum, Brazil 

**DOI:** 10.3201/eid2912.230373

**Published:** 2023-12

**Authors:** Eduardo Ferreira-Machado, Juliana Amorim Conselheiro, Bruno Emerson Bernardes da Silva, Patricia Sayuri Silvestre Matsumoto, Claúdio Luiz Castagna, Aline Nitsche, Celino Simão de Lima, Douglas Presotto, Madelline Christie Rodrigues Nunes da Silva, Ticiana Brasil Ervedosa, Pedro Enrique Navas-Suárez, Ísis Paixão de Jesus, Julia de Carvalho, Rodrigo Albergaria Ressio, Cinthya dos Santos Cirqueira, Gisely Toledo Barone, Leila del Castillo Saad, Paulo Eduardo Brandão, José Luiz Catão-Dias, Juliana Mariotti Guerra, Natália Coelho Couto de Azevedo Fernandes

**Affiliations:** Adolfo Lutz Institute, São Paulo, Brazil (E. Ferreira-Machado, T.B. Ervedosa, P.E. Navas-Suárez, I.P. de Jesus, J. de Carvalho, R.A. Ressio, C.S. Cirqueira, J.M. Guerra, N.C.C.A. Fernandes);; University of São Paulo, São Paulo (E. Ferreira-Machado, J.L. Catão-Dias, L.C. Saad, P.E. Brandão, J.M. Guerra);; Laboratory of Zoonoses and Vector-borne Diseases, Zoonoses Surveillance Division, Health Surveillance Coordination, São Paulo (J.A. Conselheiro, G.T. Barone);; Surveillance Unit in Zoonoses, Campinas, Brazil (B.E.B. Silva, C.L. Castagna, A. Nitsche, C.S. Lima);; Saint Mary's University, Halifax, Nova Scotia, Canada (P.S.S. Matsumoto);; Bosque dos Jequitibás Zoo Department of Parks and Gardens, Municipal Secretary of Public Services, Campinas (D. Presotto, M.C.R.N. Silva)

**Keywords:** rabies, viruses, Lyssavirus, opossum, *Didelphis albiventris*, Artibeus spp., marsupials, infectious diseases, Brazil, South America

## Abstract

Opossums are considered resistant to rabies. Nonhematophagous bats are reservoirs of rabies in urban areas of South America. We analyzed bats and opossums tested for rabies during 2021 in a highly urbanized city in Brazil to understand spillover in an urban setting. Wildlife surveillance is necessary to prevent rabies in humans and domestic animals.

Rabies is a viral zoonosis with high mortality rates caused by *Lyssavirus rabies* lineages (rabies virus, RABV) (*1*). Opossums of the genus *Didelphis* are marsupials widely distributed in the Americas, synanthropic in urban scenarios, and considered resistant to RABV (*2*). The main urban reservoirs of RABV in Brazil are nonhematophagous bats with distinct lineages and epidemiologic aspects (*3*). In 2021, passive surveillance programs detected an unusual case of rabies in a white-eared opossum (*D. albiventris*) by a RABV lineage of frugivorous bats of genus *Artibeus* spp. in Campinas, São Paulo state, Brazil, the 10th most urbanized city in the country (*4*). To elucidate the dynamics of this spillover, we describe the results of passive surveillance for rabies in bats and opossums in Campinas in 2021.

## The Study

In 2021, we tested samples of frozen brain tissue from 930 bats and 22 opossums for rabies by direct fluorescent antibody test and confirmed infection by virus isolation in cell culture (*5*) in Campinas. Fixed formalin brain tissue fragments in 15 of these 22 opossums were analyzed by histopathology. In addition, for the opossum that tested positive for rabies, we performed reverse transcription PCR and subsequent phylogenetic analysis of the glycoprotein gene of RABV in the frozen brain tissue and conducted immunohistochemical analysis for rabies in fixed formalin tissues (cerebrum, cerebellum, heart, lungs, liver, spleen, kidney, and adrenal glands) (Appendix 1; Appendix 2). Ethics approval was granted by the Ethics Committee in the Use of Animals of the School of Veterinary Medicine and Animal Science, University of São Paulo (approval no. 8227140222), according to the Ethical Principles in Animal Research.

**Figure 1:**
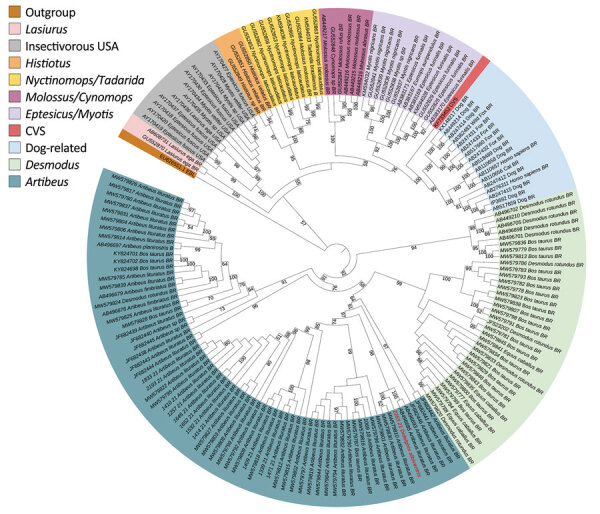
Rabies virus G gene phylogenetic tree showing specific clusters for different genera of bats in Brazil and dog-related samples in study of naturally acquired rabies in a white-eared opossum, Brazil (red text). The phylogeny was reconstructed by maximum-likelihood estimation from nucleotide sequences. Bootstrap values of >50% are depicted (1,000 bootstrap replicates). CVS corresponds to a fixed strain of the rabies virus. European bat lyssavirus-1 was used as an outgroup. The tree was visualized using iTOL version 6 (*6*). GenBank accession numbers are provided for reference sequences.

**Figure 2:**
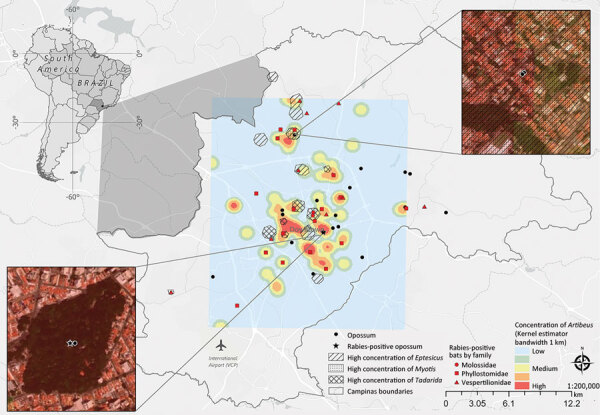
Kernel density map of concentration of *Artibeus *spp. bats in study of naturally acquired rabies in a white-eared opossum, Brazil. The kernel concentration layer of *Artibeus* is overlapped by layers of high concentration of *Eptesicus*, *Myotis*, and *Tadarida* spp. The opossum was found in a vegetated area with a high concentration of *Artibeus* spp. bats.

Of the 22 opossums tested for rabies, 1 (4.5%) adult female white-eared opossum (*D. albiventris*) had a positive result. Death was caused by traumatic lesions in 10 (45.4%) opossums; 4 (18.2%) of those deaths were caused by interspecies interactions with dogs. Of the 15 opossums analyzed by histopathology, 14 (93.3%) were found in the urban zone, inside households in densely urbanized areas, or in residences on the outskirts of the city; death was caused by traumatic lesions in 10 (66.7%) opossums, 4 of those deaths were caused by interspecies interactions with dogs. On histopathologic examination, we observed no lesions in 8 opossums, hemodynamic lesions in 4, autolysis in 2, and mononuclear meningoencephalitis in the rabies-positive opossum (Appendix 1 Figure 1). In the rabies-positive opossum, RABV antigen was detected by immunohistochemistry in the cerebrum (Appendix 1 Figure 2), cerebellum, adrenal gland, liver, and heart. The RABV-positive opossum was found in a zoo located within a park in the urban center of Campinas and demonstrated signs of the paralytic form of RABV infection. Phylogenetic reconstruction demonstrated that the RABV clustered within the frugivorous fruit-eating bats (*Artibeus* spp.) lineage circulating in Brazil (GenBank accession no. ON604858) (Figure 1).

During 2021, the frequency of rabies detected in bats was 3.2% (30/930). Among the rabies-positive bats, 17 (56.7%) were frugivorous species of fruit-eating bats (*Artibeus* spp.); the other 13 (43.4%) were insectivorous bats of *Eptesicus* spp., *Myotis* spp., and *Tadarida* spp. (Appendix 2 Table 3). In total, bats from those 4 genera represented 153 (16.4%) of the total bats investigated. Bats were tested periodically, and different seasonality peaks were noted in frugivorous and insectivorous bats (Appendix 1 Figures 5, 6). Rabies-positive bats were found in the urban perimeter of the municipality of Campinas; 73.3% were found in areas of sparse vegetation and 26.7% in areas of remnants of vegetation (Appendix 1 Table). We identified bats in a regular spatial distribution throughout the city; we observed a small area of concentration in the north and a slight concentration of rabies-positive bats in the center of the city (Appendix 1 Figure 3). According to genus classification, *Artibeus* spp. bats were found in medium and high concentrations and overlapped spatially with a high concentration of insectivorous bats. Of note, opossums were found near areas of medium to high bat concentrations, and the rabies-positive opossum was captured in a vegetated area with a high concentration of *Artibeus* spp. bats (Figure 2). We also found a spatial diffusion of *Artibeus* spp. bats that overlapped with the rabies-positive opossum (Appendix 1 Figure 4), demonstrating a time overlap in August 2021.

## Conclusions

Experimental virus inoculation in the 1960s led to initial suggestions of resistance to infection by RABV in *Didelphis* spp. opossums. (*7*). Reports of RABV in opossums are scarce; their low body temperature (34.4–36.1°C [94–97°F]) and the minimal possibility of surviving an attack by a rabid animal have been suggested as probable causes of the low prevalence of this disease in opossums in North America, where wild carnivorous mammals are natural reservoirs (*2*). Despite the low reports of rabies in opossums, a seroprevalence study conducted in São Paulo state observed a prevalence of RABV of 1.6% (5/312) in *Didelphis* spp., indicating contact between this animal population and RABV (*8*). Neurologic signs demonstrated by the rabies-positive opossum in this study are associated with paralytic form rabies, a common form transmitted by bats (*9*), and detection of viral particles in other organs indicates a phase of systemic spread. Interspecies interactions with bats in urban centers could be hypothesized as a route of RABV to the opossum, as has been observed in recent episodes of RABV in cats in Campinas (*10**,**11*). Unlike the scenario described in North America (*2*), opossums might survive interactions with bats. Opossum deaths detected in this study occurred in anthropic areas of the city; they were more prevalent in homes and were caused by traumatic events, such as attacks by dogs, warning of the possible risk for infection with RABV in domestic animals.

Frugivorous and insectivorous bats are reservoirs of RABV in urban centers of South America; bat lineages are replacing RABV canid lineage after successful vaccination efforts were adopted in Brazil in the dog population (*11*,*12*). The spatial distribution of captured bats and opossums revealed an overlap in habitats between rabies-positive bats and opossums in urban areas. The rabies-positive opossum was found in a vegetated area within a very urbanized area densely occupied by *Artibeus* spp. bats; those areas of dense bat population might create conditions in which rabies transmission, and development of new hosts and strains, is more tied to ecologic factors than to the phylogenetic characteristics of the hosts (*13*). In São Paulo state, vaccination campaigns for dogs and cats were discontinued after dog RABV lineages had not been detected for >20 years. Spillover cases such as those described in this study indicate the importance of wildlife mammal surveillance to detect RABV, particularly in urban areas, where those animals can assume the role of host and act as a source of infection for humans. Spatial analysis can be a powerful tool to assist in rabies surveillance. Although some studies have conducted mapping of bat populations in cities in Brazil (*11*,*14*), such studies are scarce and need structured surveillance programs with trained teams. In addition, we noted seasonality in the RABV bat genus and in rabies-positive bats; insectivorous bats were commonly positive in summer and spring and the frugivorous genus *Artibeus* bats were more commonly positive in fall and winter, as described by Dias et al. (*11*).

This case shows that opossums are susceptible to rabies and can potentially acquire RABV from bats, as was suggested by the ecospatial analysis. Elucidating this possibility—through the detection of the dead opossum—occurred through integrated surveillance involving motivated field and laboratory teams. Our findings highlight the need for continuous surveillance of wildlife to clarify the dynamics of zoonotic diseases and to prevent their occurrence in humans and domestic animals, in agreement with a One Health approach.

Appendix 1 Additional information about naturally acquired rabies in white-eared opossum, Brazil.

Appendix 2 Additional data from study of naturally acquired rabies in white-eared opossum, Brazil.
