# Supplementary material for: Naturally Acquired Rabies in White-Eared Opossum, Brazil
Source: Emerg Infect Dis. 2023 Dec;29(12):2541–5. doi: 10.3201/eid2912.230373 (PMC10683831; doi:10.3201/eid2912.230373)
Supplement: Appendix 1 — Additional information about naturally acquired rabies in white-eared opossum, Brazil. [file 23-0373-Techapp-s1.pdf]

Article DOI: https://doi.org/10.3201/eid2912.230373

EID cannot ensure accessibility for supplementary materials supplied by authors. Readers who have difficulty accessing supplementary content should contact the authors for assistance.

Naturally Acquired Rabies in White-Eared Opossum, Brazil

Appendix 1

Material and Methods

Material and Local of Analysis: From January to December 2021, frozen brain tissue samples of 1,049 bats and 22 opossums were investigated for rabies in Campinas by Direct Fluorescent Antibody Test (dFAT) and confirmatory by Virus Isolation in Cell Culture (VICC). Data of the animal, e.g., taxonomic classification, sex, age class, and the address in which they were found, were recorded. Of the 1,049 bats, 119 individuals were discarded from analyses for inability to rabies test by autolysis or not determined by inconsistency in the diagnostic sheet. In addition, tissue fragments fixed in formalin buffered 10% of 15 opossums were collected and the necropsy exams were compiled by data from necropsy, causa mortis, gross lesions, and the place where the animals were found... The formalin fixed paraffin embedded samples were analyzed by histopathology. The positive opossum was tested additionally to the immunohistochemistry (IHC) test for rabies, RT-PCR and sequencing of the glycoprotein gene .

dFAT and VICC: dFAT was conducted with impression smears made from sections of the brain that were fixed with cold acetone ('30'), stained with the anti-rabies antibody conjugate (in-house Pasteur Institute) in a humid chamber ('30' at 37°C) and buffered with PBS ('10'). The slides were then examined under a fluorescence microscope (Nikon EFD-3) with a 40x objective in search of fluorescent antigen-antibody aggregates. The VICC was performed with frozen samples of a 20% suspension (mass/volume) of brain tissue, and PBS was obtained by grinding with mortar and pestle and centrifuged at 1500 x g (30' at 4°C). The clarified homogenate was

then inoculated in murine neuroblastoma cells (4x105 cells/mL) in triplicate and the microplate was incubated at 37°C in a 5% CO2 humidified incubator for 96 hours. After the incubation period, the microplate was fixed and stained as described above and observed in a fluorescence microscope (Zeiss Axio Vert.A1) with a 20x objective in search of fluorescent infected cells.

Histopathology: Fixed samples were embedded in paraffin wax, sectioned at 4 μm-thick, and stained with hematoxylin and eosin for histologic analysis. For the CNS analysis, no degenerative changes, e.g., vacuolar degeneration and death neurons were considered due to the lack of knowledge between the time of death and the *postmortem* exams and collected performance.

IHC: Deparaffinized 3μm sections of tissues in silanized slides were submitted to enzymatic digestion (20' at 37°C) by proteinase K (125mg/ml). Endogenous peroxidase was blocked with 6% hydrogen peroxide (30') followed by overnight incubation with mouse hyperimmune antiserum polyclonal (in-house - Evandro Chagas Institute, Pará, Brazil) at a concentration of 1/2000. The signal was amplified by Polink-2AP Broad Kit (GBI Labs; WA; USA) for '60' conjugated to alkaline phosphatase and visualization was achieved by warp red(3′) (Polink-2 AP Broad Detection System; GBI Labs, WA, USA, cat. D24–110). The samples were counterstained with Harris Hematoxylin (20"), followed by dehydration and slide mounting with synthetic resin. Brain mammal tissue fragments known to be positive and confirmed by IHC were used as positive controls. The same steps were followed for the negative control, except for the primary antibody incubation, replaced by non-immune serum from a mouse.

RT-PCR and phylogeny: Brain samples were submitted to RNA extraction with TRIzol (Thermo Fisher Scientific) following the manufacturer's instructions. Complementary DNA synthesis was made using the Superscript VILO cDNA synthesis kit (Thermo Fisher Scientific) following the manufacturer's protocol. PCR was performed using Platinum Taq DNA Polymerase (Thermo Fisher Scientific), and the primers Ga3222–40 (5′ CGCTGCATTTTRTCARAGT 3′) and Gb4119–39 (5′ GGAGGGCACCATTTGGTMTC 3′) targeting the glycoprotein gene as described elsewhere (*1*). Gel electrophoresis was used to confirm specific product amplification. The amplicon was purified by an enzymatic method (ExoSAP-IT – Thermo Fisher Scientifc), and the BigDye Terminator v3.1 Cycle Sequencing kit (Thermo Fisher Scientifc) was used for sequencing reactions. Sequences were obtained in ABI-

3500. PHRED algorithm was used for base quality analysis of the sequence generated. Values equal to or higher than 20 were considered real. Next, the software BioEdit Sequence Alignment Editor v. 7.0.3 (*2*) was used for generating the consensus sequence and multiple alignments. A database was built with sequences retrieved from GenBank representative of rabies virus lineages circulating in Brazil, and the phylogenetic reconstruction was inferred by Maximum Likelihood using PAUP 4.0 (*3*) with GTR+I+G as an evolutionary model with 1,000 bootstrap replicates. European Bat Lyssavirus was used as an outgroup.

Geospatial Analysis: Bats and opossums locations were georeferenced using an API in Google Sheets Geocoding service (*4*). The geographic coordinates were imported into ArcGIS Pro 2.9.5, of which 930 (88.6% of bats) and 18 (81.8% of opossums) locations of animal captures were successfully located. After validation, 94 bats (8.96%) and four opossums (18.18%) had to be manually geo-edited to ensure the correct locations. Each animal represented a point (shapefile) and was categorized according to its taxonomy and outcome diagnosis in the thematic maps. Spatial analysis was performed using Kernel estimator density (*5*). We defined a bandwidth of 1,000m to identify the density of bats by genus and overlap its information with the Opossum's location. In addition, areas of influence of 50m (buffers) were created around the location of the capture of positive animals to identify the presence of vegetation, classified as densely vegetated, remnants of vegetation, sparsely vegetated, and very sparsely vegetated, ranging from higher to lower arboreal vegetation cover, respectively (see Appendix 1 Table).

References

1. Sato G, Itou T, Shoji Y, Miura Y, Mikami T, Ito M, et al. Genetic and phylogenetic analysis of glycoprotein of rabies virus isolated from several species in Brazil. J Vet Med Sci. 2004;66:747–53. PubMed https://doi.org/10.1292/jvms.66.747

2. Hall TA. BioEdit: a user-friendly biological sequence alignment editor and analysis program for Windows 95/98/NT. Nucl. Acids Symp. Ser. 1999;41:95–8 [cited 2023 Mar 2]. https://www.academia.edu/2034992/BioEdit_a_user_friendly_biological_sequence_alignment_editor_and_analysis_program_for_Windows_95_98_NT

3. Swofford D, Sullivan J. Phylogeny inference based on parsimony and other methods using PAUP. In: Lemey P, Salemi M, Vandamme A, eds. The phylogenetic handbook: a practical approach to

phylogenetic analysis and hypothesis testing. Cambridge: Cambridge University Press; 2009. p. 267–312.

4. Letunic I, Bork P. Interactive Tree Of Life (iTOL) v4: recent updates and new developments. Nucleic Acids Res. 2019;47(W1):W256–9. PubMed https://doi.org/10.1093/nar/gkz239

5. Bailey TC, Gatrell AC. Interactive spatial data analysis. New York: Longman; 1995.

Appendix 1 Table. Classification of the land use and land cover where the rabies-positive animals were investigated

Interpretation keys	Buffer	Bats	Opossums
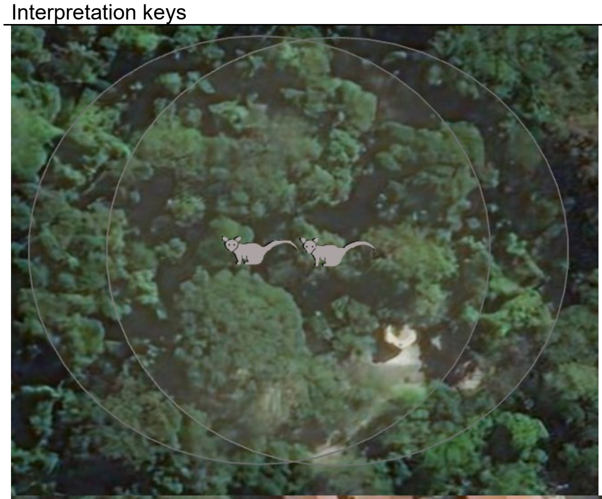	Densely vegetated	–	1 positive
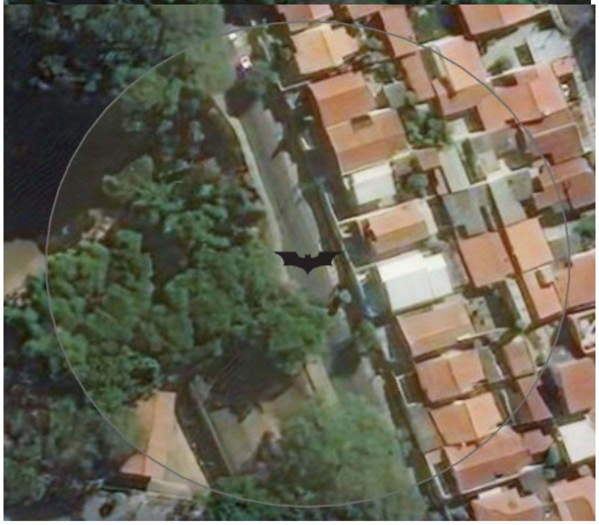	Remnants of vegetation	8 (6 *Artibeus*, 2 *Myotis*)	–

Interpretation keys	Buffer	Bats	Opossums
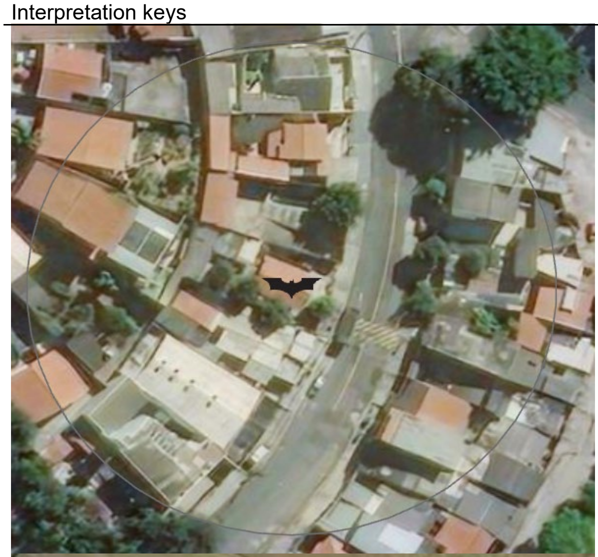	Sparsely vegetated	15 (10 *Artibeus*, 3 *Epitesicus*, 2 *Myotis*)	–
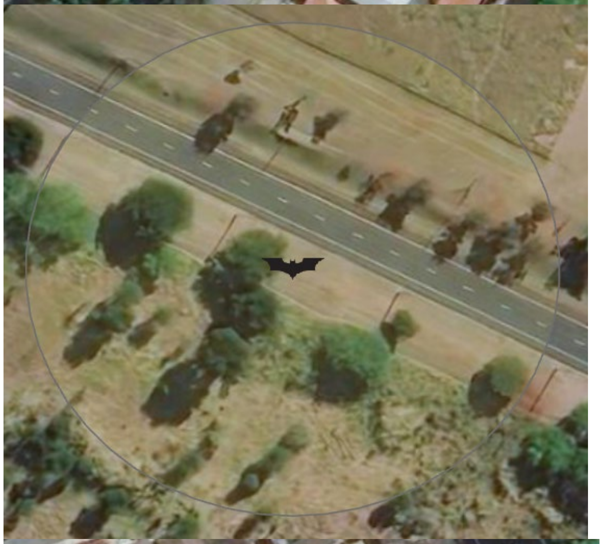			
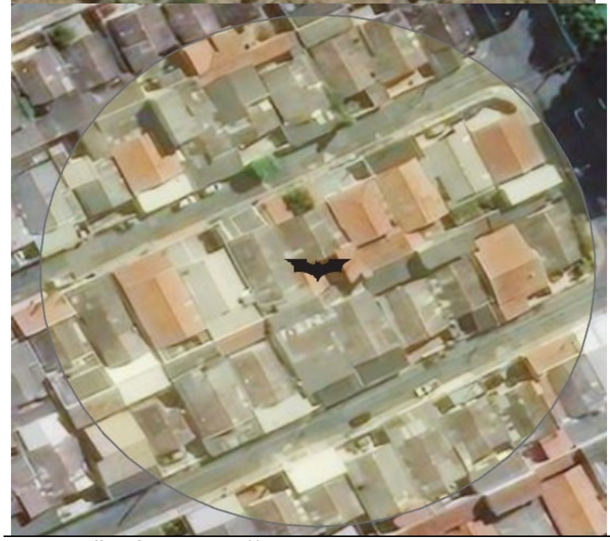	Very sparsely vegetated	7 (2 *Tadarida*, 1 *Myotis*, 3 *Epitesicus*, 1 *Artibeus*)	–

Yellow buffer of 50m with 90% transparency around the animal capture location. ArcGIS Pro 2.9.5. Imagery basemaps (Source: Maxar 2021/07/24; 0.46m spatial resolution). Cartographic scale 1:1,000.

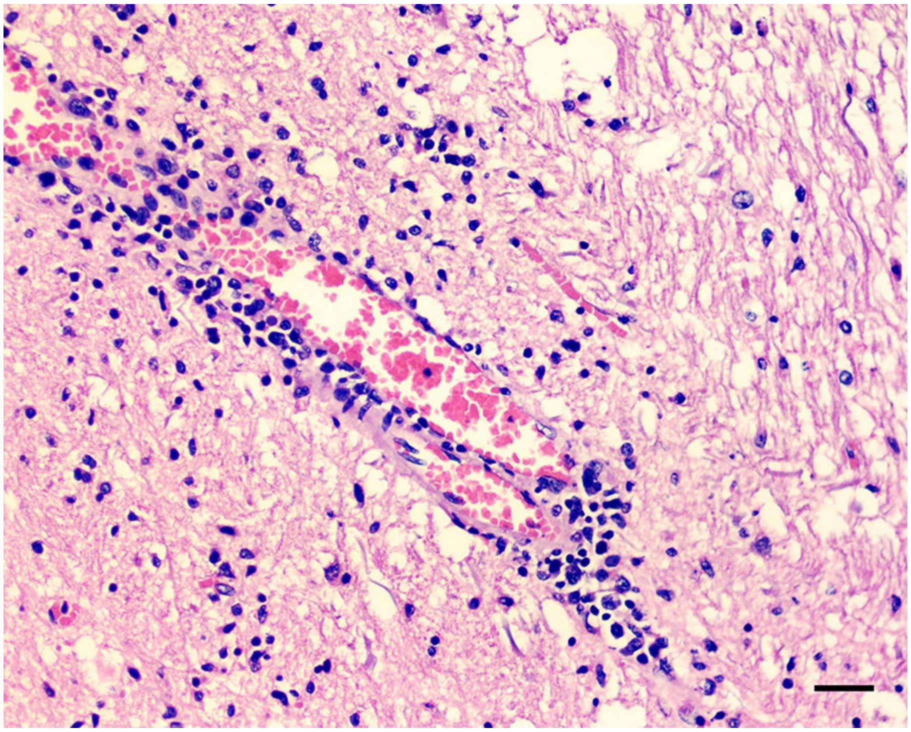

Appendix 1 Figure 1. CNS histology of rabies in opossum. HE, 200x, Cerebellum. Mononuclear perivascular cuffing.

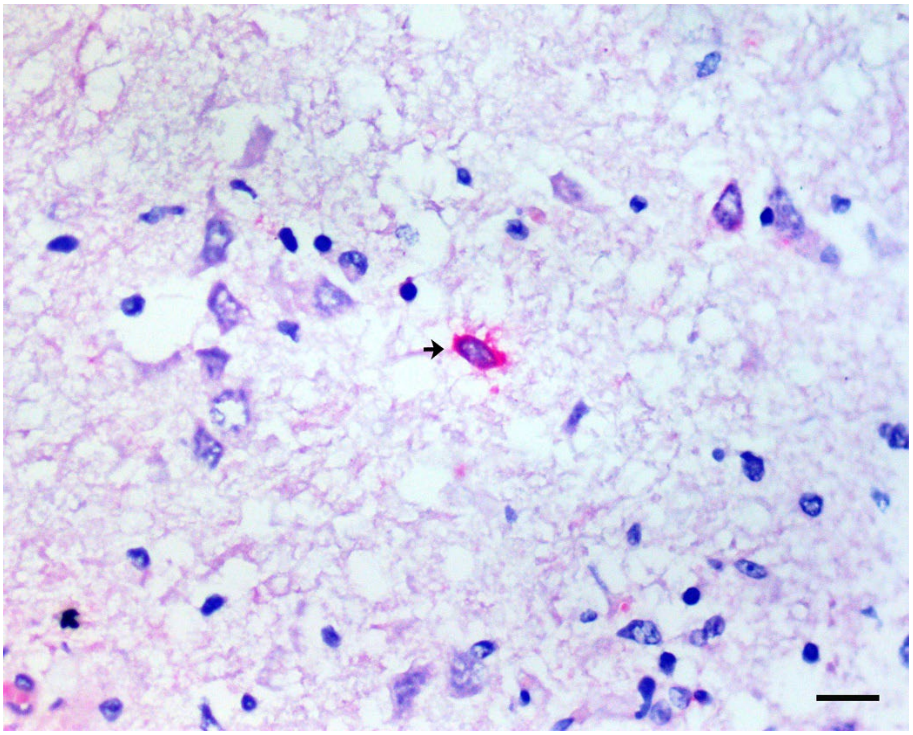

Appendix 1 Figure 2. IHC of rabies in the positive opossum. IHQ, 400x, Brain. Neuronal cell with cytoplasmic antigen detected with AP for LR (arrow).

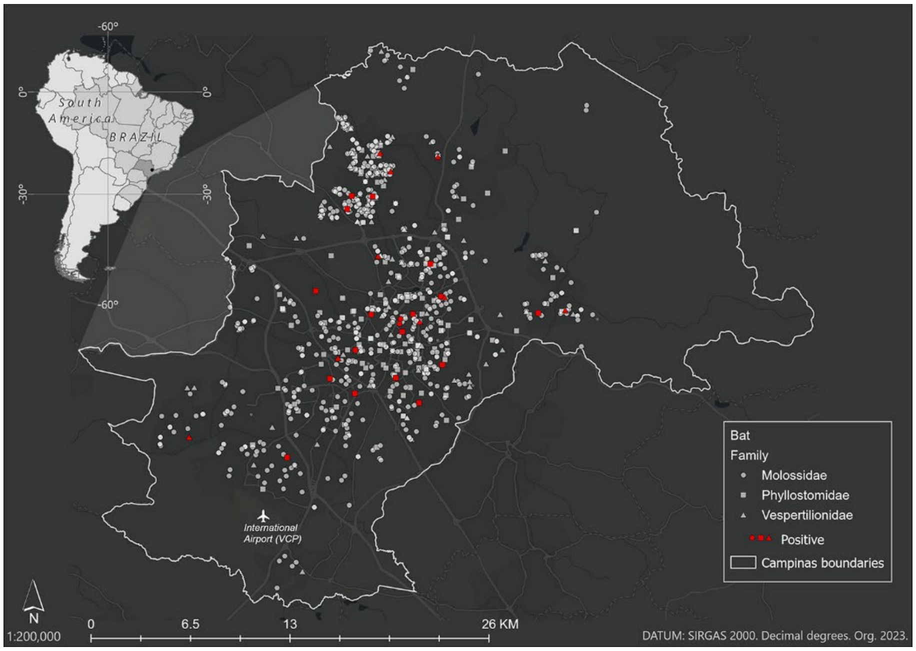

Appendix 1 Figure 3. Spatial distribution of bats classified by Family and positive diagnostic. Each gray dot represents one collection of bats: Molossidae are represented by circles, Phyllostomidae by squares, and Vespertilionidae by triangles. The positive ones are colored in red.

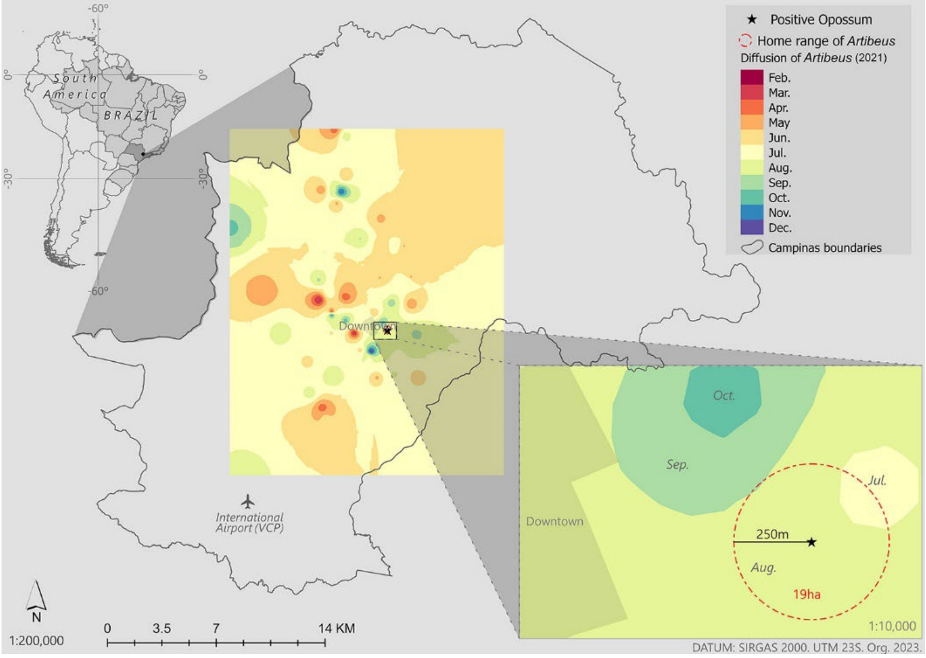

Appendix 1 Figure 4. Diffusion of *Artibeus* captured from January to December 2021. The dates were interpolated from February to December, varying from red to blue. The location where the positive opossum was found corresponds to August; however, considering the home range of Bats (≈19ha), in addition to August, the diffusion shows also July and September 2021.

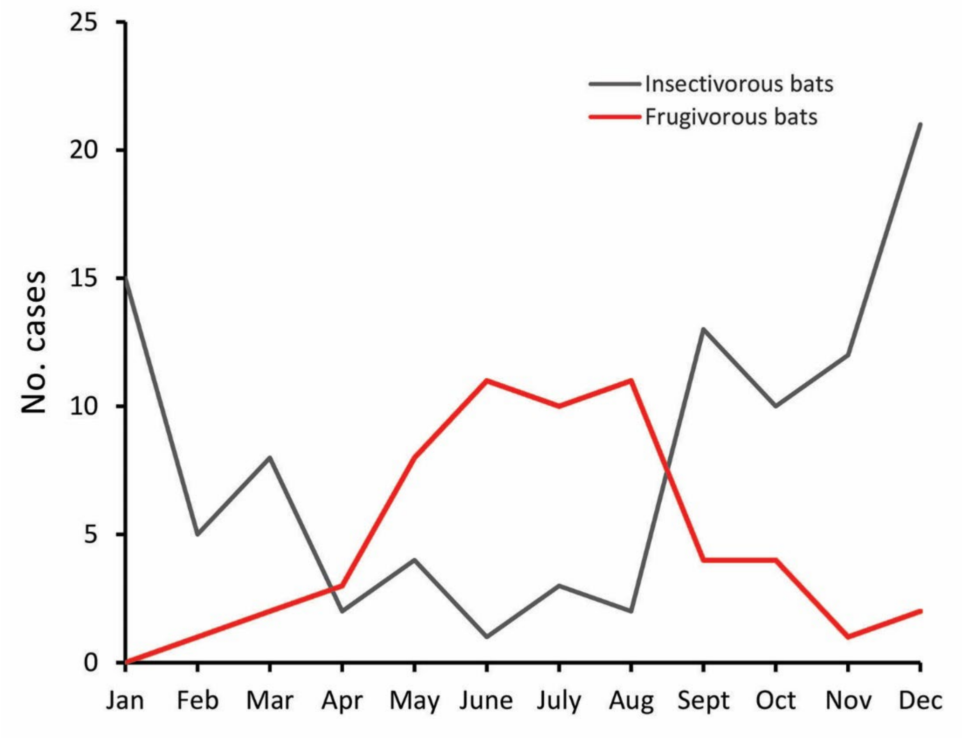

Appendix 1 Figure 5. Temporal distribution of total genus RABV detected in insectivorous and phytophagous bats.

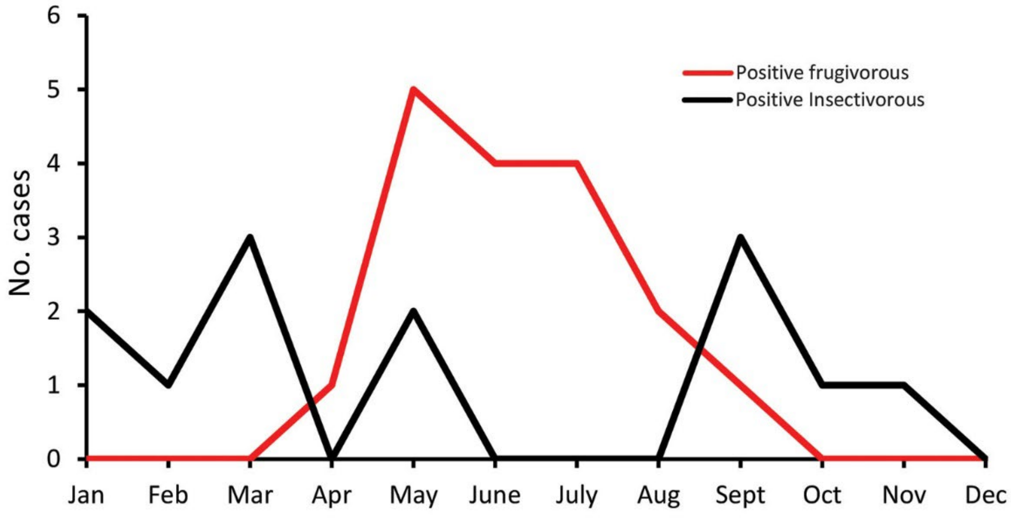

Appendix 1 Figure 6. Temporal distribution of total bats RABV detected in insectivorous and phytophagous bats.
